# Unravelling the genetic framework associated with grain quality and yield-related traits in maize (*Zea mays* L.)

**DOI:** 10.3389/fgene.2023.1248697

**Published:** 2023-08-07

**Authors:** Mehak Sethi, Dinesh Kumar Saini, Veena Devi, Charanjeet Kaur, Mohini Prabha Singh, Jasneet Singh, Gomsie Pruthi, Amanpreet Kaur, Alla Singh, Dharam Paul Chaudhary

**Affiliations:** ^1^ Division of Biochemistry, Indian Institute of Maize Research, Ludhiana, Punjab, India; ^2^ Department of Plant Breeding and Genetics, Punjab Agricultural University, Ludhiana, Punjab, India; ^3^ Department of Basic Sciences and Humanities, Punjab Agricultural University, Ludhiana, Punjab, India; ^4^ Department of Floriculture and Landscaping, Punjab Agricultural University, Ludhiana, Punjab, India; ^5^ Agricultural and Environmental Sciences, Macdonald Campus, McGill University, Montreal, QC, Canada; ^6^ Department of Biotechnology, Punjab Agricultural University, Ludhiana, Punjab, India

**Keywords:** maize, yield, quality, meta-QTLs, breeder-friendly, candidate genes

## Abstract

Maize serves as a crucial nutrient reservoir for a significant portion of the global population. However, to effectively address the growing world population’s hidden hunger, it is essential to focus on two key aspects: biofortification of maize and improving its yield potential through advanced breeding techniques. Moreover, the coordination of multiple targets within a single breeding program poses a complex challenge. This study compiled mapping studies conducted over the past decade, identifying quantitative trait loci associated with grain quality and yield related traits in maize. Meta-QTL analysis of 2,974 QTLs for 169 component traits (associated with quality and yield related traits) revealed 68 MQTLs across different genetic backgrounds and environments. Most of these MQTLs were further validated using the data from genome-wide association studies (GWAS). Further, ten MQTLs, referred to as breeding-friendly MQTLs (BF-MQTLs), with a significant phenotypic variation explained over 10% and confidence interval less than 2 Mb, were shortlisted. BF-MQTLs were further used to identify potential candidate genes, including 59 genes encoding important proteins/products involved in essential metabolic pathways. Five BF-MQTLs associated with both quality and yield traits were also recommended to be utilized in future breeding programs. Synteny analysis with wheat and rice genomes revealed conserved regions across the genomes, indicating these hotspot regions as validated targets for developing biofortified, high-yielding maize varieties in future breeding programs. After validation, the identified candidate genes can also be utilized to effectively model the plant architecture and enhance desirable quality traits through various approaches such as marker-assisted breeding, genetic engineering, and genome editing.

## 1 Introduction

Maize (*Zea mays* L.), the third most prevalent cereal crop after wheat and rice, is consumed as a staple food in many developed and developing countries worldwide (Prasanna et al., 2001). Being a C4 and day-neutral plant, maize has less water requirement and wider adaptability under a range of agro-climatic conditions (Kaur et al., 2022). Combating the ever-increasing demands for food due to the continuously increasing population is an extreme challenge for developing countries. With the emergence of the green revolution, the yield-related parameters took the whole attention of maize breeders, thus the nutritional quality remained a complex problem (Maqbool et al., 2021). Later, improvement in breeding strategies to develop nutritionally superior maize resulted in poor agronomic characteristics leading to huge productivity loss. Since the nutritional value is determined by both physiological traits and nutrient content, knowledge of the inheritance of grain yield, nutrient content (macro and micro-nutrients), as well as the genetic relationships among these traits, can facilitate maize breeders working on biofortified varieties with enhanced yield potential (Das et al., 2021).

Traits contributing to yield and grain quality are usually antagonist to each other. Moreover, such traits are quantitatively inherited and controlled by number of genetic loci. Numerous mapping studies have been conducted in maize which have led to the identification of hundreds of QTLs associated with nutritional quality traits such as protein, starch, fat, carotenoids, tocopherol, phosphorus contents, *etc.*, and yield-related traits including grain yield *per se*, kernel length, kernel width, kernel weight, *etc.* (Chen et al., 2008; Li et al., 2012; Zdunic et al., 2014; Venado et al., 2017; Fei et al., 2022; Xu et al., 2022; Banerjee et al., 2023).

The validity of QTL mapping findings is influenced by various factors, such as the specific experimental conditions, size and composition of the mapping population, density of genetic markers employed, statistical methodologies utilized, and the presence of genotype-environment and epistatic interactions (Goffinet and Gerber, 2000; Mackay, 2001; Arcade et al., 2004; Halladakeri et al., 2023). Additionally, it is common for QTLs to span relatively large genetic intervals, which can pose challenges when transferring desired QTLs through marker-assisted breeding (MAB). This phenomenon is often referred to as “linkage drag,” where undesirable genetic regions linked to the target QTL are unintentionally transferred along with it. To overcome this limitation, it is essential to precisely localize QTLs within narrow genetic intervals. This not only minimizes the potential for linkage drag but also enhances the efficiency and effectiveness of MAB and QTL cloning efforts. To enhance the reliability and robustness of QTL studies, researchers often employ multiple mapping populations evaluated across diverse locations and over multiple years. This approach helps account for genotype-environment interactions, ensuring that the identified QTLs exhibit consistent effects across different genetic backgrounds and environmental conditions. Consensus QTLs that consistently demonstrate significant associations with the target trait across multiple populations and environments are regarded as particularly suitable candidates for integration into MAB programs (Goffinet and Gerber, 2000; Halladakeri et al., 2023).

Meta-QTL analysis represents a conceptual approach that combines QTL discoveries from various studies and refines the positions of QTLs on a consensus map (Goffinet and Gerber, 2000). When numerous QTLs associated with the specific trait are analysed together through meta-analysis, the resulting consensus QTLs are referred to as “meta-QTLs (MQTLs).” This approach can not only identify redundant QTLs and candidate genes (CGs), but also facilitate the selection of promising QTLs for breeding applications involving MAB (Goffinet and Gerber, 2000; Sandhu et al., 2021; Saini et al., 2022; Sheoran et al., 2022; Tanin et al., 2022; Kumar A. et al., 2023; Halladakeri et al., 2023; Karnatam et al., 2023).

In maize, significant advancements have been made in the field of MQTL analysis, which involves identifying genetic regions associated with various traits such as fungal disease resistance (Gupta et al., 2023), multiple abiotic stress tolerance (Sheoran et al., 2022), popping traits (Kaur et al., 2021), and root-related traits (Karnatam et al., 2023). Additionally, specific MQTL studies have focused on yield-related traits (Semagn et al., 2013; Wang Y. et al., 2016; Wang et al., 2020; Chen et al., 2017; Zhou et al., 2020) or quality-related traits (Jin et al., 2013; Dong et al., 2015). However, it is important to note that none of the previous studies have conducted MQTL analysis for QTLs associated with both yield-related and quality traits in maize. Additionally, several new studies on QTL analysis for both yield-related and quality traits in maize have been conducted since the publication of earlier studies, but they have not been utilized for predicting MQTLs or identifying promising candidate genes (CGs). In our present study, we aimed to address this gap by simultaneously investigating the genetic regions influencing both yield-related and quality traits in maize. As a result, we can contribute to the development of improved maize varieties with enhanced yield and quality traits, benefiting both farmers and consumers alike.

The current study utilized QTL data from 56 studies associated with various yield-related and quality traits to perform MQTL analysis. The primary objectives of this study were as follows: i) constructing a high-density consensus genetic map, ii) identifying robust and consistent MQTLs with narrower confidence intervals compared to initial QTLs, iii) determining precise flanking markers for MQTLs to facilitate MAB, iv) validating MQTLs through genome-wide association studies (GWAS), v) identifying CGs within promising MQTL regions, and vi) conducting expression analysis of the identified CGs. It is anticipated that these efforts will greatly enhance the selection efficiency for various yield-related and quality traits in maize.

## 2 Materials and methods

In the present analysis, a MQTL analysis was conducted to identify the genomic regions associated with yield and quality-related traits in maize. The analysis involved following five key steps: i) bibliographic search and compilation of QTL mapping studies related to quality and yield-associated traits, ii) integration of high quality linkage maps and markers from the individual studies to create a consensus map, iii) prediction of MQTLs and their validation using GWAS, iv) mining genes within potential MQTL regions and *in silico* expression analysis, v) synteny analysis among the maize, wheat, and rice for the key genomic regions. By employing these steps, the study aimed to gain insights into the genetic basis underlying both yield and quality-related traits in maize and related cereals.

### 2.1 Bibliographic search and compilation of QTL mapping studies

The various traits that impact both maize quality and yield are illustrated in Supplementary Table S1. Several QTL mapping studies have highlighted the significance of these traits in defining maize quality and enhancing yield. To gather relevant information, these studies were collected from online platforms such as Google Scholar (https://scholar.google.com/) and PubMed Central (https://www.ncbi.nlm.nih.gov/pmc/), using appropriate keywords. The collected studies provided essential data for the meta-QTL analysis, including the size and type of the mapping population, the specific map used in each study, the marker positions associated with QTLs, the traits linked to the QTLs, the confidence interval of the markers, the LOD score, and the phenotypic variation explained by individual QTLs (represented as R^2^ or PVE). However, certain studies were not included in the meta-analysis due to the lack of important data. Further, to facilitate clarity and uniqueness, the QTLs used in the present study were assigned distinctive names, which included the trait name, followed by the chromosome number, and finally, the number of the QTL on the chromosome.

### 2.2 Consensus map development and QTL projection

To construct a highly dense consensus map, the present study incorporated two high-quality, high resolution, mixed marker (involving RFLP, SSR, SNP and InDels) linkage maps, i.e., “ISU Integrated IBM 2009” and IBM2 (intermated B73/Mo17) (https://www.maizegdb.org/data_center/map) These maps were carefully compiled and enriched to ensure accuracy and reliability. Furthermore, all the markers flanking the QTLs extracted from different studies were integrated into this consensus map, facilitating the projection of a greater number of QTLs. The construction of the consensus map was accomplished using the LPMerge package within the R environment as described by Kumar R et al (2022).

The collected QTLs underwent a rigorous screening process to identify reliable QTLs with complete information essential for projection and meta-analysis. Our initial selection criteria involved identifying stable and consistent QTLs across diverse environments within each study, while excluding those that were specific to particular environments (i.e., environment-specific QTLs). Additionally, QTLs without available LOD or PVE values were excluded, as well as those lacking information on flanking markers and genetic positions. Subsequently, the selected QTLs were projected onto the consensus map using BioMercator v4.2.3 software, which served as the projection tool (ChardonVirlon et al., 2004). For QTL projection optimization, QTLProj, a dynamic strategy, was employed. This strategy utilizes pairs of common markers flanking the QTLs in the original map, along with an estimate of the distance between the initial and consensus maps. The QTL projection procedure and map distance calculation are influenced by the minimum value of the flanking marker distance ratio and the minimum *p*-value. These parameters were carefully adjusted to ensure the similarity of flanking marker interval distances between the initial and consensus maps, thereby enhancing the accuracy of QTL projection.

### 2.3 Prediction of MQTLs

In this study, maize quality and yield-related traits were considered as meta-traits. A meta-QTL refers to a genomic region that contains two or more QTLs for a trait, obtained from at least two different mapping studies. The prediction of meta-QTLs was carried out using a two-step process described by Veyrieras et al. (2007). In the first step, the best MQTL model was selected by comparing five different models: i) AIC (Akaike information criterion), ii) AICc (AIC correction), iii) AIC3 (AIC 3 candidate models), iv) BIC (Bayesian information criterion), and v) AWE (average weight of evidence). The model with the lowest value among three out of the five models was chosen. The second step involved determining the total number of MQTLs on a chromosome, their weightage according to the selected model, and the confidence interval (CI).

To calculate the LOD and PVE values of the predicted MQTLs, the average of the LOD and PVE values of the initial QTLs comprising the meta-QTL was taken. In cases where multiple MQTLs were predicted on a single chromosome; a standardized naming procedure was followed. Each meta-QTL was assigned a name starting with “MQTL,” followed by the numeric representation of the chromosome number, a period, and another numeric value indicating the sequence of the MQTL on that specific chromosome (e.g., MQTL1.1, MQTL1.2). To obtain the physical position of each meta-QTL, the flanking markers associated with them were used. Published studies or online databases such as MaizeGDB (https://www.maizegdb.org/), GrainGenes (https://wheat.pw.usda.gov/GG3/), and Gramene (https://www.gramene.org/) were utilized to obtain information on the nucleotide sequences of the flanking markers. The obtained sequence information was then subjected to BLAST analysis against the reference genome of maize available on the Ensembl Plants database (https://plants.ensembl.org/index.html).

### 2.4 Validation of MQTLs using GWAS

To validate the projected MQTLs, a validation process was conducted using 10 independent GWAS studies focused on traits related to grain yield and quality (*viz.*, Li et al., 2013; Liu et al., 2016; Fang et al., 2021; Ma and Cao, 2021; Ndlovu et al., 2022; Qu et al., 2022; Suwarno et al., 2015; Zheng et al., 2021; Zeng et al., 2022; Zhang et al., 2022). These GWAS studies encompassed maize populations consisting of 180–410 genotypes/accessions. To determine the physical position of the identified significant markers (marker-trait associations; MTAs) from the GWAS studies, the database MaizeGDB was utilized. The retrieved physical positions were then compared to the physical positions of the MQTLs present on individual chromosomes. MQTLs that co-localized with markers from the significant GWAS studies were considered as GWAS-verified MQTLs, further enhancing their credibility and relevance in the context of the study.

### 2.5 Selection of breeder friendly MQTLs

The MQTLs investigated in this study cover broad genomic regions impacting yield and quality related traits in maize. To enable practical implementation by plant breeders, “breeder friendly MQTLs” were selected based on the following criteria: PVE >10%, genetic confidence interval <2 cM, physical confidence interval <1 Mb, and involvement of at least 10 initial QTLs. These precise MQTLs provide targeted regions of interest for plant breeders to enhance grain yield and quality in maize varieties.

### 2.6 Candidate gene mining within breeder friendly MQTL regions

The regions identified as BF-MQTLs were further investigated for candidate gene mining and expression analysis related to maize quality and yield traits. MaizeGDB, utilizing the MaizeMine tool, was employed for candidate gene mining. The genomic regions corresponding to each MQTL were analysed to identify genes of interest within the Zm-B73-REFERENCE-NAM-5.0 assembly. This approach provided valuable information about the various genes encompassed within the selected genomic regions.

### 2.7 Expression analysis of candidate genes and their syntenic regions in wheat and rice genomes

Each gene identified within the MQTL region was analysed for expression patterns using the qTeller tool available on MaizeGDB. qTeller provides information on gene expression across different tissues throughout maize development. To gain insights into the functional characteristics of the identified genes, as well as to explore potential orthologous relationships, functional descriptions and orthologous studies in wheat (*Triticum aestivum*) and rice (*Oryza sativa*) were conducted using the MaizeMine tool on MaizeGDB. The wheat and rice homologous collected through MaizeMine were assessed through Ensembl Plants to retrieve their chromosomal location on respective genomes. MQTLs containing similar gene models located on conserved genomic regions across wheat, rice, and maize were considered as ortho-MQTLs.

## 3 Results

### 3.1 Bibliographic search and collected QTL information

A comprehensive analysis was conducted, encompassing data from 56 interval mapping studies, yielding a total of 2,974 QTLs associated with 169 component traits, with 48 yield-associated traits, 23 oil-associated traits, 28 protein-associated traits, 13 starch-associated traits, 25 carotenoid-associated traits, 16 metal ion associated traits, 10 tocopherol associated traits and 6 traits deciphering associated macromolecules (Supplementary Table S1). Among these studies, 37 studies focused on quality-associated traits, 10 on yield-related traits and 9 studies had a combined focus on both yield and maize grain quality (Supplementary Table S2). The studies employed 67 different bi-parental populations, including doubled haploid (DH), recombinant inbred lines (RILs), F_2_, and backcross populations. It is worth noting that certain populations were assessed for various traits in multiple studies. The range of population sizes utilized in the study varied from 10 to 4,699 individuals (Supplementary Table S2).

**FIGURE 1:**
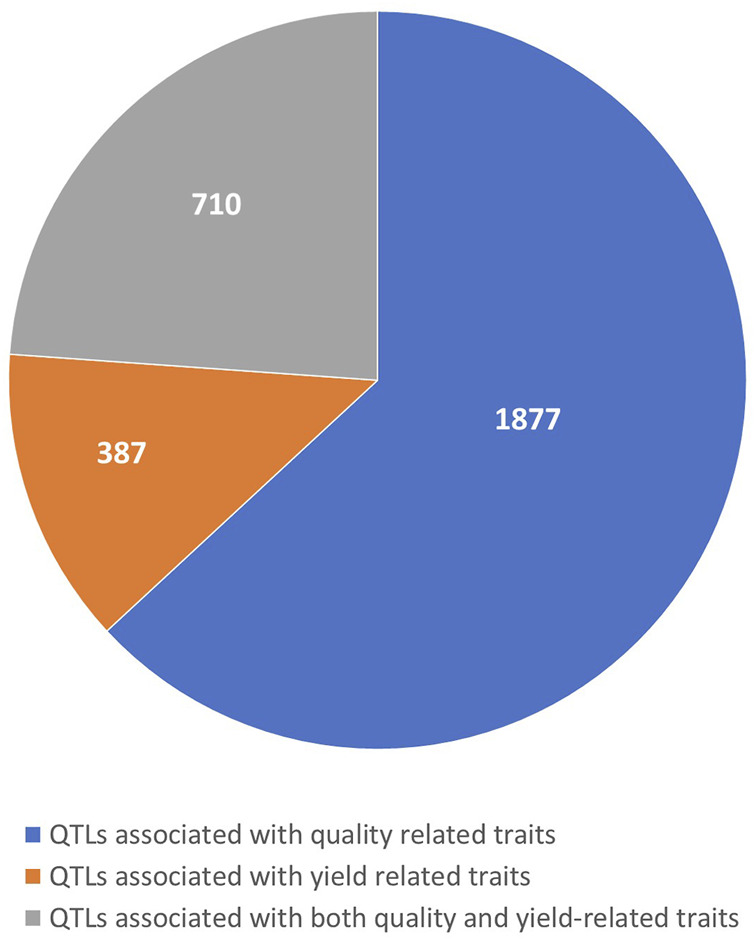
Distribution of QTLs associated with quality and yield related traits.

The number of QTLs varied across different categories of traits, with 1,877 QTLs identified for quality-related traits, 387 QTLs for yield-related traits, and 710 QTLs for combined quality and yield-related traits (Figure 1). The distribution of collected QTLs across the 10 maize chromosomes was uneven, ranging from 190 QTLs on chromosome 10 to 377 QTLs on chromosome 1, with an average of approximately 284 QTLs per chromosome. The individual QTLs exhibited a wide range of LOD scores, spanning from 1.88 to 75.3, and averaging at 4.71. Notably, over 80% of the QTLs had LOD scores falling within the 3 to 75.3 range. As for the PVE, it varied from 0.01% to 49.3%, with an average of 9.01%. These QTLs demonstrated a typical L-shaped distribution, with approximately 70% of them showing a PVE of 10% or less. Regarding the confidence intervals (CIs) of the individual QTLs, they ranged from 0 to 119.5 cM, with an average of 11.31 cM. Interestingly, a considerable portion of the QTLs (more than 38%) had CIs equal to or greater than 10 cM.

### 3.2 Features of consensus map

The consensus map integrated an extensive set of 57,523 markers, encompassing a diverse range of SSR, RFLP, and SNP markers. These markers were strategically distributed across a genetic span of 3,959.9 cM. Across the individual chromosomes, the lengths exhibited notable variation, ranging from 157.4 cM for chromosome 10 to 1,135.4 cM for chromosome 1, with an overall average of 395.99 cM. Remarkably, an average of approximately 14 markers were identified per cM, emphasizing the marker-rich nature of the map. Examining the marker density across chromosomes, the maximum density of 34.3 markers per cM was observed on chromosome 5, signifying a concentrated marker distribution. Conversely, chromosome 7 displayed the minimum marker density of 8.9 markers per cM, indicating a relatively sparser marker distribution on that particular chromosome.

### 3.3 QTLs projected on consensus map

**FIGURE 2:**
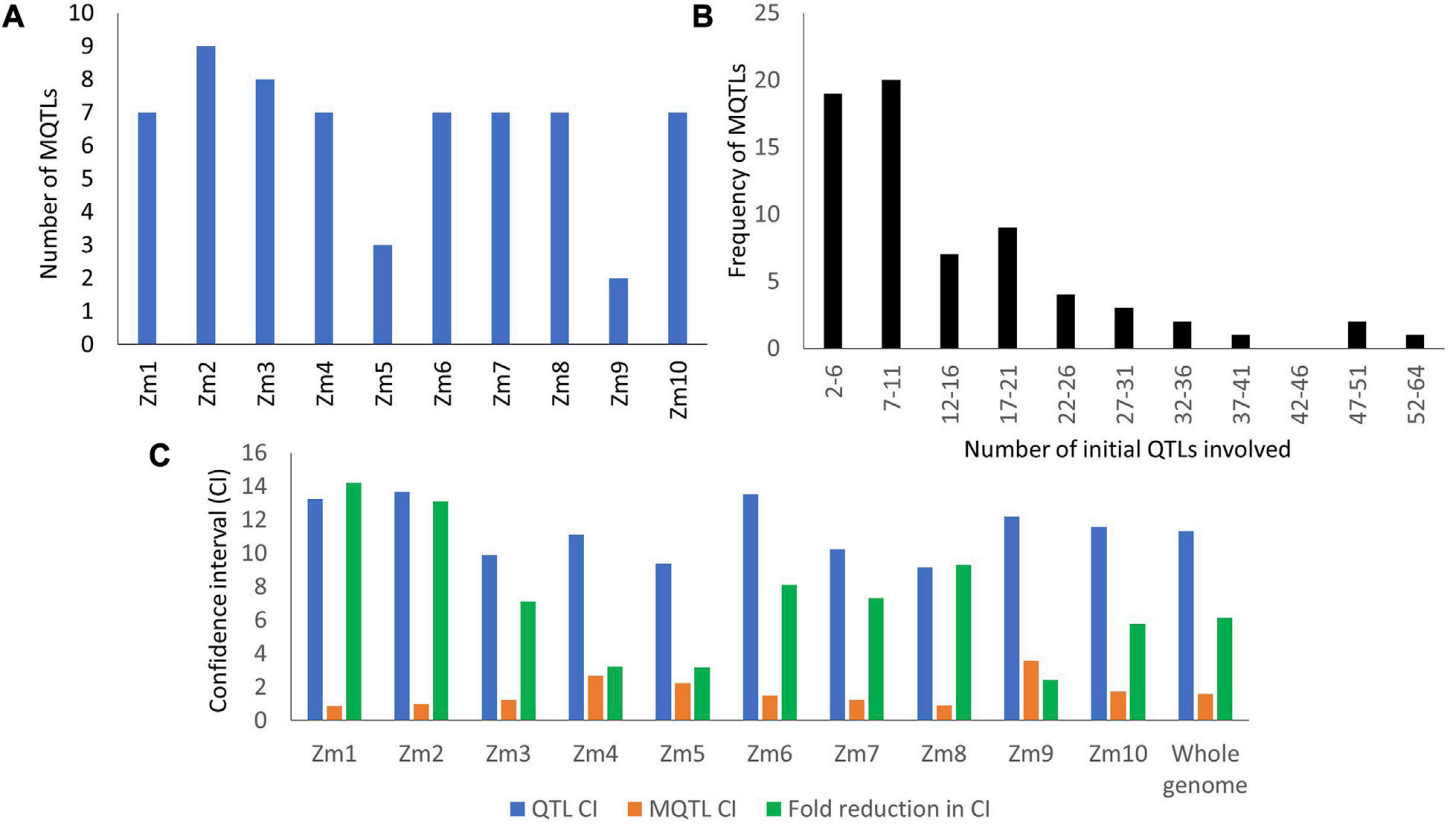
Some characteristic features of QTLs and MQTLs **(A)** Distribution of MQTLs on different maize chromosomes; **(B)** Number of QTLs under each MQTL; **(C)** Average CI of QTLs and MQTLs available on different maize chromosomes.

Among the QTLs selected from the collected QTLs, as many as 965 were successfully projected onto the consensus map. The 965 projected QTLs were grouped into 75 hotspot regions. However, seven hotspot regions consisting of only a single QTL from an individual study were not considered true MQTLs. As a result, a total of 68 MQTLs were obtained from the present study, distributed across the 10 chromosomes. The highest number of MQTLs, nine in total, were identified on chromosomes 2 and 7, while the minimum of two MQTLs were found on chromosome 9 (Figure 2A). The number of QTLs within each MQTL varied, ranging from 2 (MQTL 3.9) to 64 (MQTL 1.1). Sixteen MQTLs comprised 20 or more QTLs from different studies (Figure 2B). The proportion of MQTLs and the number of QTLs varied on each chromosome (Supplementary Table S3).

All the MQTLs identified in this study exhibited LOD scores ranging from 3 to 12.25 and phenotypic variation explained (PVE) ranging from 3.86% to 21.41%. Among the reported MQTLs, 19 had high LOD scores (≥5) and PVE values (≥10%), namely,: MQTL1.4, MQTL2.4, MQTL2.8, MQTL3.5, MQTL5.3, MQTL6.4, MQTL6.8, MQTL7.2, MQTL7.3, MQTL7.4, MQTL7.5, MQTL7.7, MQTL7.8, MQTL8.1, MQTL8.3, MQTL8.4, MQTL8.6, MQTL10.7, MQTL10.8 (Figure 2B). The average CI of the MQTLs was 1.59 cM, which was 6.12 times smaller than the average CI of the initial QTLs (11.32 cM). Significant reductions in CI were observed on different chromosomes, with the highest fold change observed on chromosome 1 (14.21-times) and the lowest on chromosome 9 (2.4-times) (refer to Figure 2C).

**FIGURE 3:**
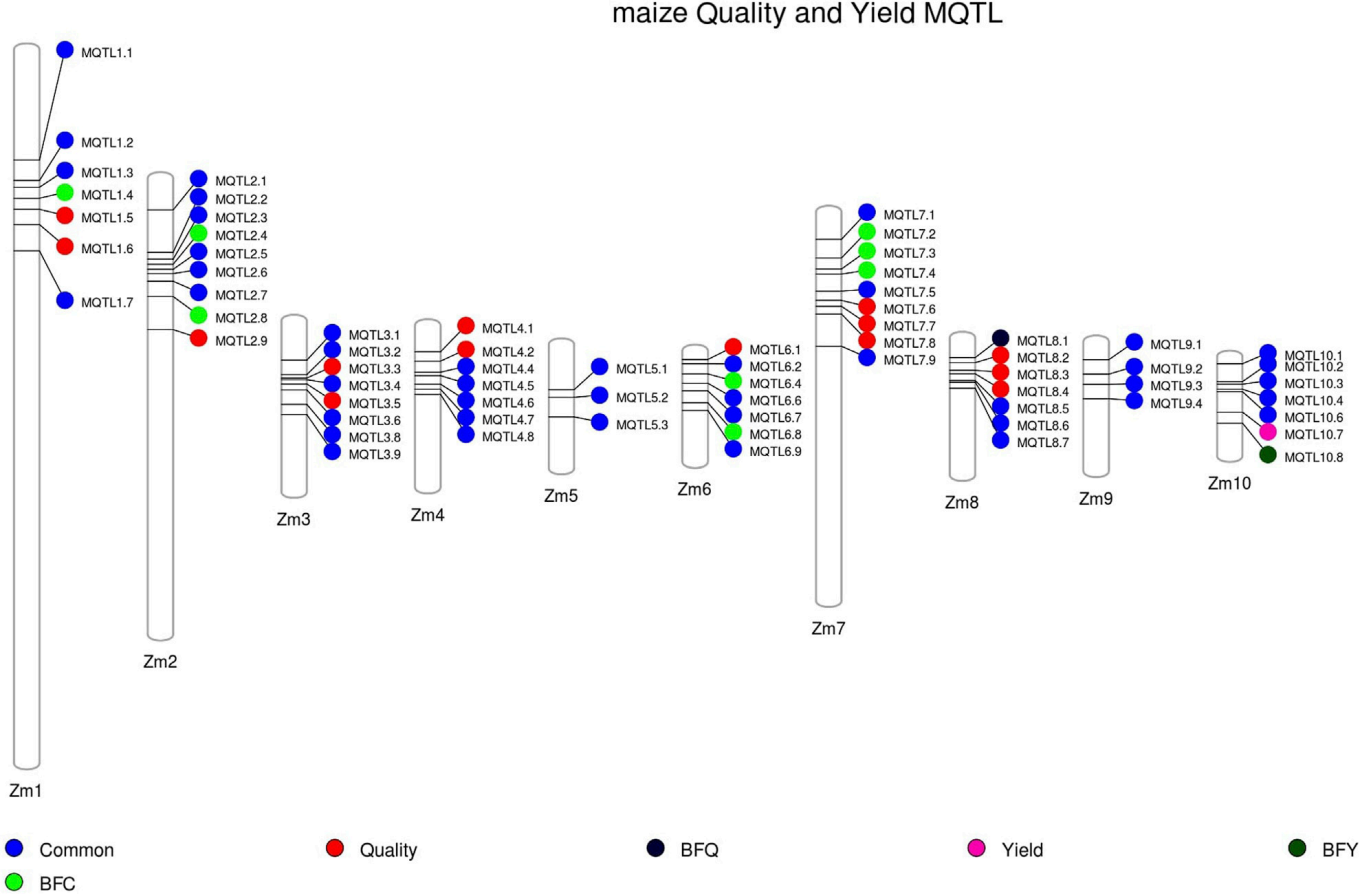
Distribution of MQTLs on different maize chromosomes. Common: MQTLs associated with both quality and yield-associated traits, BFQ: breeder-friendly quality trait MQTLs, BFY: breeder-friendly yield trait MQTLs, BFC: breeder-friendly common MQTLs (involving both quality and yield-related traits).

All the MQTLs were physically mapped onto the maize reference genome Zm-B73-REFERENCE-NAM-5.0. The physical CI of these MQTLs ranged from 2,049 bp (MQTL7.3) to 39.95 Mb (MQTL 4.4), with a mean physical CI of 3.30 Mb. Collectively, these MQTLs occupied a physical length of 224.52 Mb across the genome (refer to Figure 3). Among the 68 MQTLs, two were exclusively associated with yield related traits (MQTL10.7, MQTL10.8), and 14 were exclusively associated with quality traits (MQTL1.5, 1.6, 2.9, 3.3, 3.5, 4.1, 4.2, 6.1, 7.6, 7.7, 7.8, 8.1, 8.2, 8.3, 8.4). Notably, MQTL6.1 was found to be associated with carotenoid synthesis, while MQTL4.2 was exclusively associated with amino acid biosynthesis (Figure 3).

### 3.4 Breeders friendly MQTLs

Out of the 68 MQTLs reported, a subset of 10 MQTLs was selected and designated as “breeder-friendly MQTLs” (BF-MQTLs). The selection criteria for identifying BF-MQTLs were as follows: a PVE value of more than 10%, a genetic CI of less than 2 cM, a physical CI of less than 1 Mb, and involvement of at least 10 initial QTLs from different studies. Detailed information on these BF-MQTLs can be found in Table 1. Among the 10 BF-MQTLs, eight MQTLs encompassed QTLs associated with both quality and yield-related traits, while one MQTL included QTLs solely for quality-related traits, and another MQTL comprised QTLs exclusively associated with yield-related traits (refer to Figure 3).

**TABLE 1:** Detailed information of Breeder Friendly MQTLs. These MQTLs were selected based on the following criteria: PVE>10%, CI < 2 cM, and number of initial QTLs involved> 10.

MQTL name	chr	CI (95%)	Average pve	Average lod	No of QTLs involved	QTLs involved
MQTL1.4	1	1.03	10.02	5.30	21	qSTAR1, qINCY1-1, qSC8-1, qGP1-4, qGP1-1, qMgC1, qKD1, qZnC1-1, qATPH1-1, qED1, qBCP1-1, qED1-1, qBCT1-1, qCT1-2, qMnC1-3, qRPE1, qRPE1-1, qGY1, qKL1, qPAC1, qPH1-4
MQTL2.4	2	0.05	10.71	4.79	13	qKW2-1, qSC2-9, qSC2-3, qPC2-2, qPC2, qEF1A2, qSC2-1, qZEA/LUT/BCAR2, qZEA2, qZEX2-1, qLINO2, qOC2-5, qILE/TAA2
MQTL2.8	2	0.86	12.62	3.94	23	qSPC2-1, qEWLR2-1, qMET/TAA2, qLYS/ATT2, qOSC2, qEW2-1, qKV2-1, qOPC2-1, qGP2-5, qKV2, qOLE2-1, qMET/ATT2, qPOC2, qEEWR2-1, qLIO2, qSOC2-1, qSOC2, qEO2-2, qSTE2, qKWI2, qGDR2, qGDR2-2, qGDR2-1
MQTL6.4	6	0.78	12.87	4.66	23	qGDR6-1, qSTE6, qOC6-2, qOPC6, qOC6-4, qOLE6, qOC6-1, qLIO6, qKWI6-5, qSC6-1, qOSC6, qEO6, qBEH6, qKO6, qLIG6, qSER/TAA6, qEOD6, qKT6, qOC6-3, qHKW6, qOLE6-1, qPAL6, qTC6
MQTL6.8	6	0.93	14.12	5.94	11	qPC6-1, qPAC6, qOPAC6, qACAR6-1, qHKW6-1, qED6, qVITR6, qSPC6, qBELI6, qTEXT6, qPSC6
MQTL7.2	7	0.31	14.06	8.14	20	qHKW7, qBCRP7, qZEA7, qERN7-1, qLUT7, qZEA/LUT/BCAR7, qBCAR7, qGT/TAA7-1, qVITR7, qEF7, q100GW7, qGLX/GT7, qKV7-1, qST/TAA7, qTCAR7, qPHE7, qC/CL7, qGP7-2, qGLX/GT7-1, qGP7-6
MQTL7.3	7	0.5	10.47	4.79	32	qGP7-4, qGP7-7, qKW7-8, qKW7-7, qARG/TAA7, qKW7-2, qILE/BCAA7, qLYS/TAA7, qKW7, qLEU/BCAA7, qST/TAA7-1, qGLX7-1, qTHR/TAA7, qAT/TAA7, qKV7, qKW7-10, qLIO7, qOLE7, qKW7-5, qKW7-4, qSFA7, qFeC7-1, qLYS/TAA7-1, qGP7-1, qKW7-1, qKW7-6, qKW7-3, qKW7-9, qGP7-8, qGP7-5, qPT/TAA7, qSC7
MQTL7.4	7	0.54	10.44	5.00	41	qHKW7-1, qHKW7-3, qEL7, qKWI7, qKWI7-1, qKV7-3, qKV7-2, qKT7, qPHE/TAA7, qTCAR7-1, qCP7-1, qZEA7-1, qBCF7-1, qZnC7, qPAL7, qFAA7, qEW7, qASX/ATT7-1, qILE/ATT7, qGLY/TAA7, qPT/TAA7-1, qASX/ATT7, qARG/GT7, qLEU/BCAA7-1, qVAL/AT7, qAT7-7, qLEU/AT7, qBCAA7, qGLY/ST7-1, qCYS/TAA7, qGLX/TAA7, qSER/ST7, qLYS/ATT7, qALA/AT7, qLEU/TAA7, qLEU/TAA7-1, qVAL/BCAA7, qAT/TAA7-1, qVAL/TAA7, qPHE/TAA7-1, qPAC7
MQTL8.1	8	1.71	10.72	5.95	20	qSPC8, qTCAR8-2, qBCRY-TCAR8, q13ZBCAR8-1, qBCRY-PVA8, qPVA8-2, qTCAR8-1, qBCAR-BCRY8-2, qCBCAR8, q9ZBCAR8-1, qGTPH8, qATPH8, qTTPH8, qBCAR8-1, qDTPH8, qSTE8, qEO8, qBCRY-TCAR8-1, qPVA8-1, q13ZBCAR8
MQTL10.8	10	0.72	21.41	5.52	28	qKV10-4, qKV10-6, qKV10, qKV10-20, qKV10-17, qKV10-18, qKV10-16, qKW10-6, qKV10-13, qKV10-14, qKV10-11, qKV10-5, qKV10-7, qKW10-8, qKW10-10, qKW10-9, qKV10-19, qKW10-4, qKV10-15, qKW10-3, qKV10-12, qKV10-10, qKV10-3, qKL10, qKV10-2, qKW10-1, qKW10-5, qKWI0

### 3.5 Validation of MQTLs with the GWAS based MTAs and the availability of known genes within MQTL regions

The physical positions of MQTLs identified through meta-analysis were compared with the physical positions of significant markers (MTAs) identified for the same traits using the GWAS approach. Data obtained from 10 different GWAS studies provided information on 250 MTAs, that overlapped with 47 MQTLs out of 68 reported MQTLs. The count of overlapping MTAs or adjacent MTAs (within a 1 Mb region surrounding the MQTL regions) varied for each MQTL, with MQTL9.3 exhibiting the highest number of overlaps with 36 MTAs. Notably, among the overlapped MTAs, 35 were associated with oil-related traits, 26 were associated with yield-related traits, 17 were associated with carotenoid-related traits, 2 were associated with sugar-related traits, and 1 was associated with protein-related traits (see Supplementary Table S4 and Supplementary Figure S1). It is important to mention that these GWAS-validated MQTLs also included all the breeder-friendly MQTLs (BF-MQTLs).

GWAS studies provided valuable insights into the genes associated with the traits under investigation (see Supplementary Table S4). Specifically, a total of 13 genes were found to be co-localized with MQTLs affecting yield. These included one gene associated with cob weight (i.e., *Zm00001d002000*), two genes associated with kernel length (*viz.*, *Zm00001d036024*, *Zm00001d000184*), three genes associated with kernel number per row (*viz.*, *GRMZM2G049091*, *GRMZM2G138067*, *GRMZM2G446921*), one gene associated with kernel thickness (i.e., *Zm00001d041972*), two genes associated with kernel weight (*viz.*, *GRMZM2G010933*, *GRMZM2G464985*), one gene associated with moisture content (i.e., *GRMZM2G069024*), and four genes associated with grain yield (*viz.*, *GRMZM2G391473*, *GRMZM2G125557*, *GRMZM5G845736*, *GRMZM2G151649*). Interestingly, three breeder-friendly MQTLs (BF-MQTL2.8, BF-MQTL7.3, BF-MQTL7.4) were found to be associated with these reported genes that influence grain yield.

Furthermore, 13 genes were identified to be co-localized with some MQTLs affecting grain quality. Among them, 10 genes were associated with grain oil content (*viz.*, *GRMZM2G059138*, *GRMZM5G828253*, *GRMZM2G118423*, *GRMZM2G176542*, *GRMZM2G125268*, *GRMZM2G122767*, *GRMZM2G417435*, *GRMZM2G169089*, *GRMZM2G077789*, *GRMZM2G136072*), two genes were associated with starch content (*viz.*, *GRMZM2G404453*, *GRMZM2G104325*), and one gene was validated for grain protein content (i.e., *GRMZM2G049681*). Two breeder-friendly MQTLs (*viz.*, BF-MQTL6.4, BF-MQTL6.8) were found to be associated with these reported genes affecting grain quality. Notably, these genes encode essential enzymes (e.g., cytochrome c oxidase copper chaperone 1, serine/threonine-protein kinase D6PKL1, beta-glucosidase 11) involved in significant metabolic pathways and transcription factors (e.g., transcription factor MYBS3, transcription initiation factor IIF beta subunit), as well as proteins (e.g., protein LURP-one-related 5, F-box domain containing protein) that are believed to play crucial roles in influencing grain yield and quality (see Supplementary Table S4).

### 3.6 *In silico* gene mining and tissue-specific expression analysis

**FIGURE 4:**
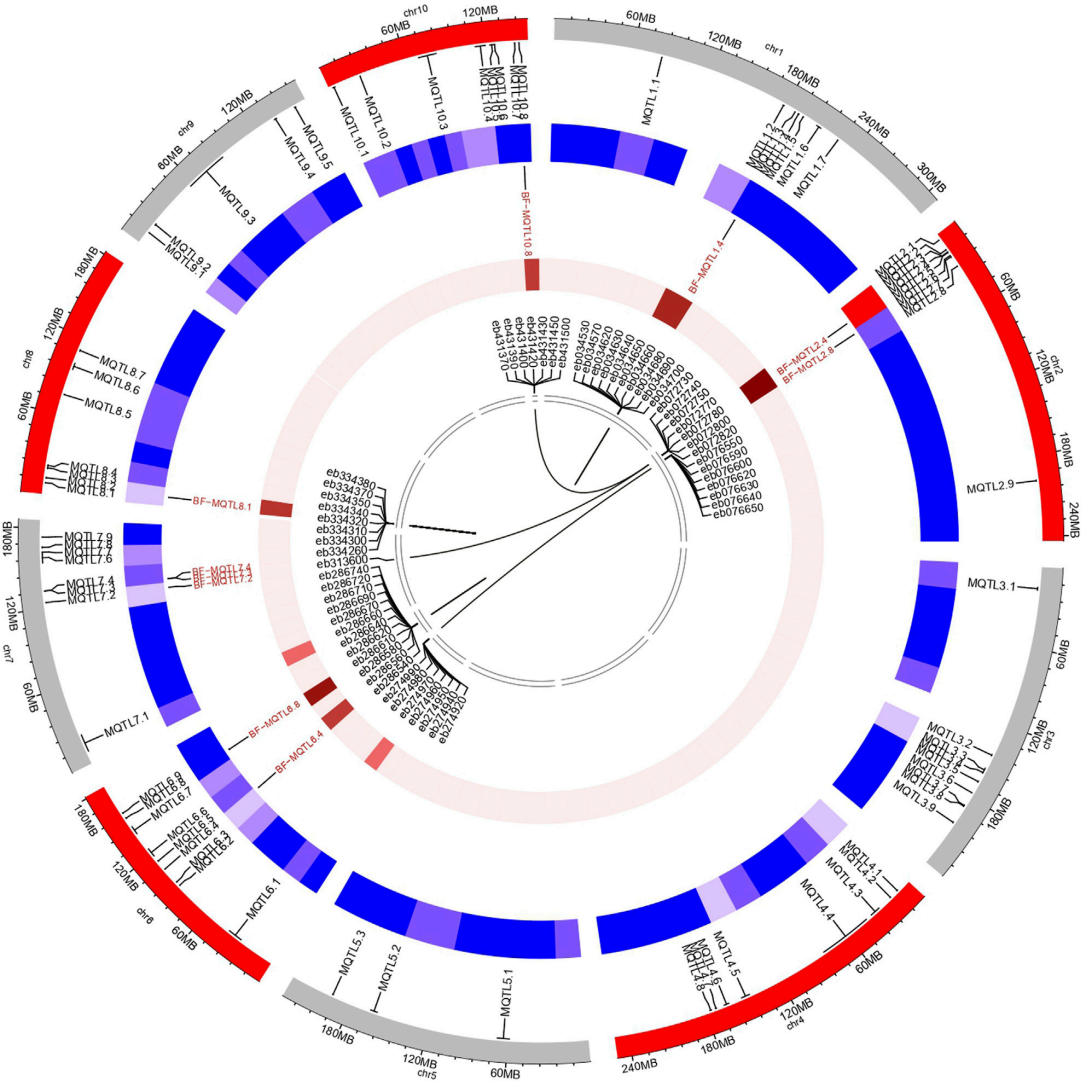
Diagram deciphering the salient characteristics of MQTLs, BF-MQTLs, candidate genes. Outermost circle represents the location of MQTLs predicted during the present study. Second circle (blue) provides an overview of density of MQTLs on different chromosomes. Third circle represents locations and densities of BF-MQTL along with putative gene density. Lines within innermost circle represents phylogenetic relationships between identified genes.

**FIGURE 5:**
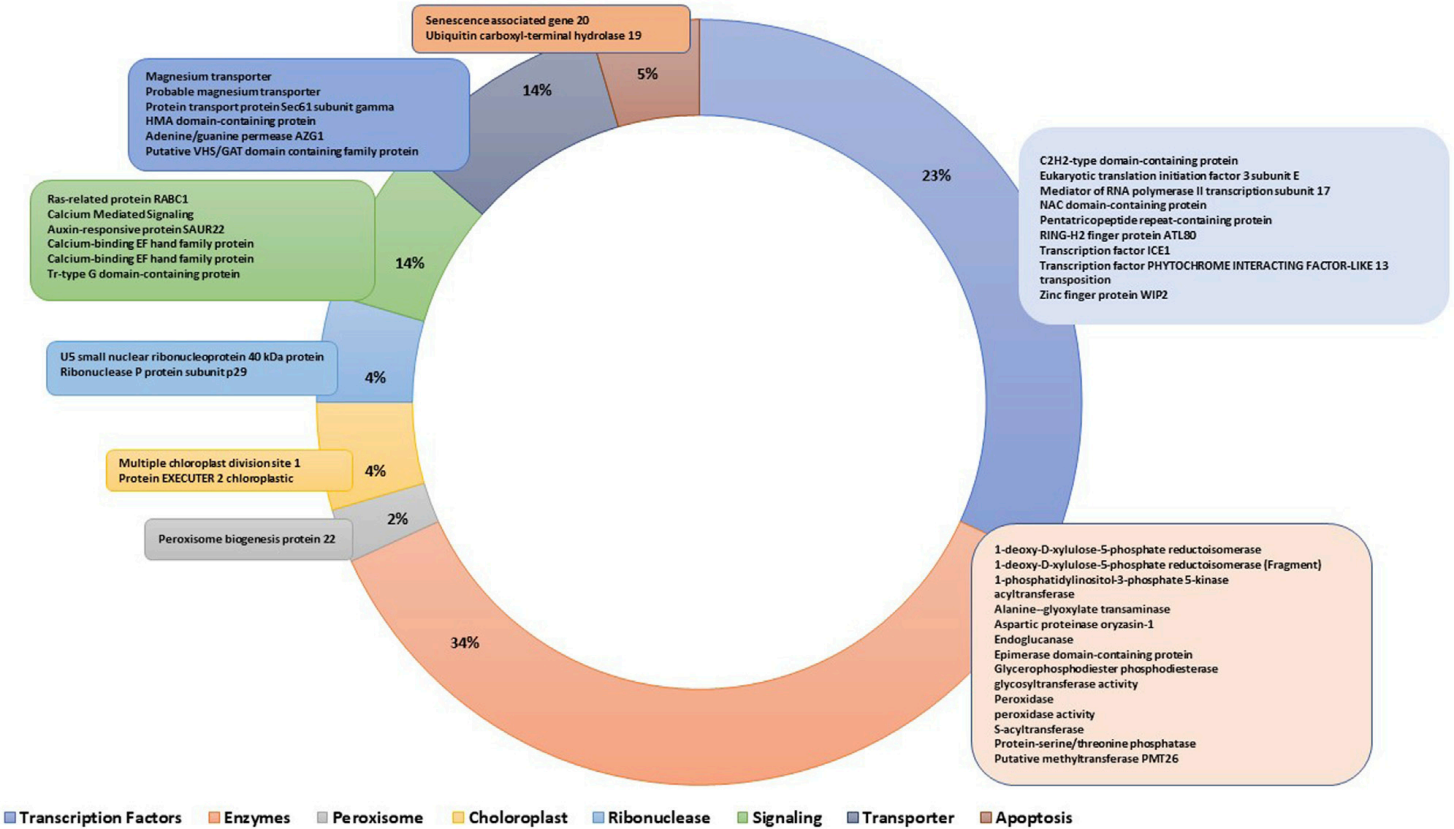
Biological functions of various classes of genes annotated under BF-MQTLs region.

The genes residing within the genomic regions of BF-MQTLs were investigated using maizeGDB database. A total of 59 genes were identified within these BF-MQTL regions, which are located on chromosomes 1, 2, 6, 8, and 10 (see Figure 4). Among the analysed genes, 15 genes were found to encode enzymes involved in crucial metabolic processes such as lipid, amino acid, protein, sugar, nucleotide and secondary metabolite metabolism. Some enzymes were also involved in isomerization, endomembrane homeostasis and gene regulation, 10 genes encoded versatile groups of transcription factors (e.g., C2H2-type domain-containing protein, eukaryotic translation initiation factor 3 subunit E, Mediator of RNA polymerase II transcription subunit 17, NAC domain-containing protein, RING-H2 finger protein ATL80, transcription factor ICE1, transcription factor PHYTOCHROME INTERACTING FACTOR-LIKE 13, transposition associated factors, zinc finger protein WIP2), 6 genes encoded transporter proteins (e.g., magnesium transporter (2), protein transport protein Sec61 subunit gamma, HMA domain-containing protein, adenine/guanine permease AZG1, putative VHS/GAT domain containing family protein, 6 genes were associated with signalling (e.g., ras-related protein RABC1, calcium mediated signalling (3), auxin-responsive protein SAUR22, tr-type G domain-containing protein), 2 genes each were related to apoptosis (e.g., senescence associated gene 20, ubiquitin carboxyl-terminal hydrolase 19), ribonuclease (e.g., U5 small nuclear ribonucleoprotein 40 kDa protein, ribonuclease P protein subunit p29), and chloroplast (e.g., multiple chloroplast division site 1, protein EXECUTER 2 chloroplastic) proteins, and one gene was reported to function in peroxisomes (i.e., peroxisome biogenesis protein 22) (see Figure 5).

**FIGURE 6:**
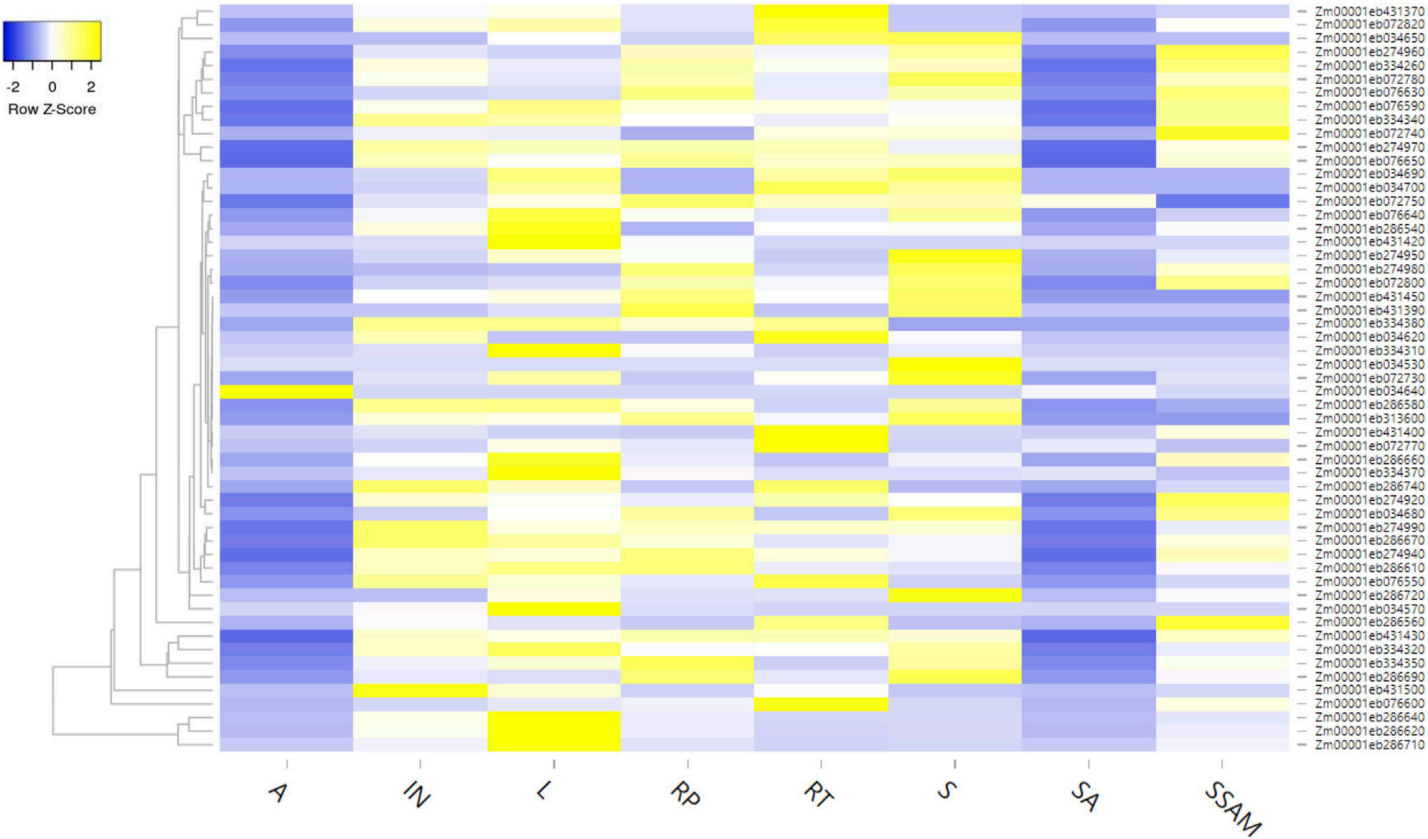
Tissue-specific expression of different genes available from MQTL regions (A: anther, IN: internode, L: leaf, RP: reproductive, R: root, S: shoot, SA: shoot axis internode, SSAM: stem and shoot apical meristem).

Additionally, the expression analysis of these genes was performed using the expression analysis tool within the MaizeMine, revealing tissue-specific expression patterns (Figure 6; Supplementary Table S5). Among the eight different tissues studied, a single gene showed expression in anthers (i.e., *Zm00001eb034640*), four genes were expressed in shoot axis internodes, 41 genes were expressed in stem and shoot apical meristems, 49 genes were expressed in reproductive tissues, 52 genes were expressed in roots and internodes, 53 genes were expressed in leaves, and 54 genes were expressed in seeds.

### 3.7 Synteny of genes identified in maize MQTL regions with wheat and rice genomes

**FIGURE 7:**
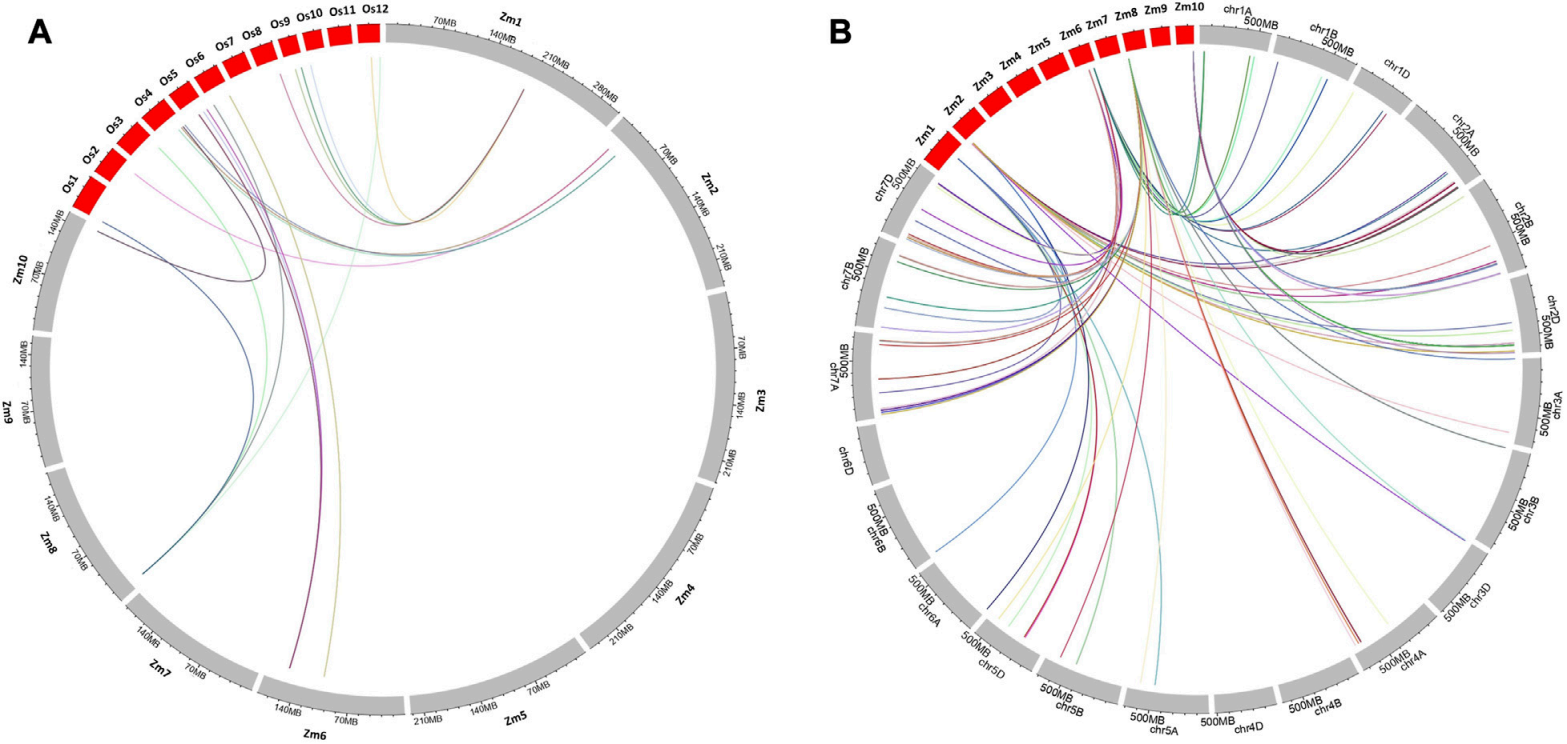
Syntenic relationships of genes available from the maize MQTL regions with the **(A)** rice and **(B)** wheat genomes. The physical lengths of the chromosomes are indicated by the rulers drawn above on each chromosome.

Synteny analysis was conducted to identify conserved regions of the reported maize MQTLs in the genomes of wheat and rice. In total, 59 genes identified in the maize genome within the 10 BF-MQTL regions corresponded to 42 genes in the rice genome (Figure 7A) and 150 genes in the wheat genome (see Figure 7B). The genes obtained from the BF-MQTLs showed variation in the number of orthologs in the wheat genome, with the maximum number of orthologs (34) observed for genes derived from MQTL10.8, while the minimum (12) was observed for genes from MQTL2.4. Similarly, in the rice genome, the maximum number of orthologous genes was reported for genes derived from MQTL1.4, while the minimum (3) was observed for genes from MQTL2.8. Overall, the synteny analysis demonstrated the presence of conserved genomic regions between maize and rice, as well as between maize and wheat. These regions can be considered as ortho-MQTL regions (see Figure 7).

## 4 Discussion

Grain quality and grain yield are critical traits in maize breeding, as they determine the nutritional value and productivity of the crop. Maize is a major source of macronutrients such as protein, starch, and fat, as well as micronutrients and phytochemicals including tocopherols, carotenoids, metal ions (Fe, Zn, *etc.*), and phytic acid, which are essential for the maize-consuming population (Owens et al., 2014; Tanumihardjo et al., 2019). Numerous studies have been conducted over the years, identifying a large number of QTLs associated with various traits related to maize grain quality and yield (Supplementary Table S1). However, most of the QTLs reported in previous studies have low PVE values, wide CIs and other potential limitations, limiting their potential for utilization in breeding programs aimed at developing nutrient-rich, high-yielding maize varieties. Moreover, several studies have demonstrated the co-localization of QTLs linked to various aspects of grain quality and yield-related characteristics in maize (Yang et al., 2013). These findings suggest that a single genetic locus may exert influence over multiple facets of grain development. Consequently, investigating these genomic regions and analysing the hotspot areas that affect both grain quality and yield can significantly enhance future maize breeding programs.

Meta-analyses of QTLs associated with a variety of traits have been recently conducted in different crops such as wheat (Kumar et al., 2021; Kumar et al., 2022 A. C.; Kumar et al., 2023 S.; Saini et al., 2021; Saini et al., 2022; Tanin et al., 2022), rice (Sandhu et al., 2021; Kumari et al., 2023), barley (Akbari et al., 2022), common bean (Shafi et al., 2022), pigeon pea (Halladakeri et al., 2023), including maize (Kaur et al., 2021; Sheoran et al., 2022; Wang et al., 2022; Gupta et al., 2023; Karnatam et al., 2023), for diverse traits, including both yield-related traits (Semagn et al., 2013; Wang Y. et al., 2016; 2020; Chen et al., 2017; Zhou et al., 2020) and quality traits (Jin et al., 2013; Dong et al., 2015). However, there is currently no comprehensive study on the genomic regions influencing both grain quality and yield in maize. This study aims to fill this gap by identifying QTL clusters in defined regions using a large dataset of yield-related and quality trait data from multiple studies.

The findings of this study can significantly contribute to advancing maize breeding programs. Further, our study represents a significant advancement over previous meta-analyses conducted on various yield and quality-related traits. It offers a more up-to-date and comprehensive analysis, distinguishing itself in several key aspects (Supplementary Table S6). The present study offers a broad perspective on recent mapping studies (published up to 2022) related to maize grain yield and quality. The consensus map used in this study is comprehensive and densely populated, covering the entire maize genome with high precision. A total of 57,523 markers, including SSR, SNP, and RFLP markers, were employed in the consensus maps, distributed across a length of 3,959.9 cM. The highest number of markers was reported on chromosome 1 (10,341), while the lowest was observed on chromosome 10 (4,566). The distribution of markers and QTL density on maize chromosomes observed in this study aligns with findings from previous meta-QTL studies on maize (Sheoran et al., 2022; Wang et al., 2022).

A total of 75 hotspot regions were distributed across the 10 chromosomes in the present study. The majority of these hotspot regions were found in the upper terminal regions of the chromosomes, which have been previously identified as regions with high gene density (Ghaffari et al., 2013). These regions exhibited multiple yield and quality-associated QTLs within a single MQTL region, indicating a strong correlation of these genomic regions in enhancing maize grain quality and yield. The hotspot regions established in present study can be considered in future breeding programs to obtain high yield and high-quality hybrids. Several biochemical and mapping studies have been conducted previously to establish correlation between yield and quality associated traits (Zheng et al., 2021; Amegbor et al., 2022).

In the context of maize, grain nutrient quality is primarily determined by the levels of tryptophan and lysine in the maize protein. Standard maize varieties generally lack sufficient amounts of these essential amino acids, resulting in reduced overall protein quality. This deficiency has led to a significant focus on quality protein maize breeding over the past decade (Prasanna et al., 2001; Prasanna et al., 2020). Maize grain quality-associated traits are complex, and they are regulated by multiple major genes and lots of minor genes. It is reported that there is a negative association between grain yield and protein quality. Tryptophan content showed a significant positive genotypic association with moisture, oil, and fiber content, but a strong negative correlation with protein and starch content (Amegbor et al., 2022). Given the intricate interplay between various quality and yield-related traits, gene pyramiding is strongly recommended to develop biofortified, high-yielding hybrids (Zheng et al., 2021). Despite several mapping studies, the goal of achieving both high yield and grain quality has proven challenging, with fluctuations observed under varying environmental conditions. The common hotspot regions for both grain yield and grain quality traits (BF-MQTL1.4, 2.4, 2.8, 6.4, 6.8, 7.2, 7.3, 7.4) can be beneficial for future breeding programs for targeting associations between yield and quality traits. Although, the significant inverse correlations observed between various traits along with the presence of shared or linked QTLs and epistasis with opposing effects, may pose potential challenges for plant breeders aiming to simultaneously enhance these traits in maize.

The present study also refined the CIs of different QTLs for various quality and yield traits, resulting in an average reduction of 6.12 times compared to the initial QTLs. Further, GWAS was employed as a precision technique to validate the reported MQTLs, with approximately 62% of the MQTLs being validated through previous GWA studies (Suwarno et al., 2015; Ma and Cao, 2021; Zheng et al., 2021; Ndlovu et al., 2022; Zeng et al., 2022; Zhang et al., 2022). MQTL9.3 showed the highest overlap with a maximum number of MTAs in GWA studies, with 36 MTAs identified. Additionally, 27 MQTLs overlapped with only one MTA. Similar results of MQTL validation through MTAs have been reported in previous studies, with validation rates of 54.6% and 63% (Saini et al., 2021; Saini et al., 2022).

The MQTLs validated through GWAS were further characterized as breeder-friendly based on their high PVE and low CI values. Among the 10 validated BF- MQTLs in the present study, a total of 59 CGs were reported. Supplementary Table S7 presents the orthologs of these 59 CGs in model crops, including *Arabidopsis* and rice, along with their respective function descriptions. Notably, some of these orthologs have been extensively studied and their associations with the traits under study have been well-documented. Among these BF-MQTLs, five (BF-MQTL 1.4, 2.4, 2.8, 6.4, 6.8) exhibited QTLs associated with both grain quality and yield. Among these promising BF-MQTLs, MQTL 6.8 had the highest number of associated genes (12), followed by MQTL1.4 with 10 genes, and 7 genes each for MQTL2.4, MQTL2.8, and MQTL6.4. Based on these findings, BF-MQTL1.4, 2.4, 2.8, 6.4, and 6.8 appear to be the most promising candidates for improving both grain yield and quality. However, further investigations are required to identify functional variants of these genes within the MQTL regions.

Several genes identified within the validated hotspot regions encode enzymes involved in various metabolic processes, such as terpenoid biosynthesis (1-deoxy-D-xylulose-5-phosphate reductoisomerase) (Zhang et al., 2020), auxin dependent endomembrane homeostasis (1-phosphatidylinositol-3-phosphate 5-kinase (Lo et al., 2022), oil biosynthesis (acyltransferase) (Yan et al., 2018), photorespiration (alanine--glyoxylate transaminase which is known to facilitate the transamination between L-alanine and glyoxylate to produce pyruvate and glycine) (Liepman et al., 2019), lipid metabolism (aspartic proteinase oryzasin-1) (Alexandrov et al., 2009), carbohydrate metabolism (endoglucanase) (Jiao et al., 2017), sugar isomerization (epimerase domain-containing protein), phosphorus remobilization (glycerophosphodiester phosphodiesterase) (Wang J. et al., 2021), *etc.*

In addition to enzymes, some genes also encode for potent transcription factors. An example of such transcription factors is the C2H2-type domain-containing proteins, known as zinc finger proteins, which play a pivotal role in regulating plant growth and development (Li et al., 2022). Eukaryotic translation initiation factor 3 subunit E is involved in translating specific mRNA subsets related to cell proliferation. Mediator of RNA polymerase II transcription subunit 17 acts as a coactivator, regulating the transcription of almost all RNA polymerase II-dependent genes. NAC domain-containing proteins have diverse functions, including nuclear localization, DNA binding, and the formation of homodimers or heterodimers with other NAC domain-containing proteins (Nuruzzaman et al., 2013). Moreover, certain NAC-TFs, such as *ZmNAC128* and *ZmNAC130*, have been identified to play crucial roles in the accumulation of starch and protein in maize, as highlighted in a study by Zhang et al. (2019). Pentatricopeptide repeat-containing proteins participate in RNA regulation and metabolism within plant organelles (Wang X. et al., 2021), and they are recognized for their multifaceted roles in plant growth and development (Li et al., 2022). The RING-H2 finger protein ATL80, featuring zinc finger domains, plays a role in stress response (Gao et al., 2012) and has been associated with influencing plant architecture and grain yield in rice (Yan et al., 2022). Another transcription factor, ICE1 MYC-like bHLH, acts as a transcriptional activator during cold stress response (Waititu et al., 2021) and is also known to enhance yield in rice. TF PHYTOCHROME INTERACTING FACTOR-LIKE 13 participates in light-induced transcription and plays a role in plant growth and internode elongation (Wu et al., 2019). Additionally, there are transposition-associated genes and the Zinc finger protein WIP2, which have been linked to plant growth (Wang et al., 2010; Wang B. et al., 2016; Li et al., 2022).

A few genes reported within the MQTL regions were categorized as peroxisome biogenesis protein 22 that participates in peroxisome assembly (Jiao et al., 2021), multiple chloroplast division sites 1 that leads to natural variation in chloroplast size (Kadirjan-Kalbach et al., 2019), protein EXECUTER 2 chloroplastic (Zhang et al., 2019), U5 small nuclear ribonucleoprotein (part of spliceosome), ribonuclease P protein subunit p29 which is involved in Mg2 -dependent hydrolysis (Ohtani, 2018). Several genes are implicated in transport across bio-membranes, for instance, the putative VHS/GAT domain-containing family protein is known to be involved in protein transport and energy metabolism, and its overexpression has been reported to enhance biomass (Nunes et al., 2019). Additionally, the magnesium transporter is recognized for its role in chlorophyll synthesis (Li et al., 2018; Li, 2020), while the protein transport protein Sec61 subunit gamma is responsible for mediating endoplasmic reticulum translocation (Zimmermann et al., 2011). Furthermore, the HMA domain-containing protein contains a conserved domain found in several heavy metal transport or detoxification proteins (Li et al., 2018; Cao et al., 2019), and the Adenine/guanine permease *AZG1* plays a crucial role in facilitating adenine and guanine transport across membranes.

Two genes were associated with apoptosis involving senescence-associated gene 20, and ubiquitin carboxyl-terminal hydrolase 19 (Zhang et al., 2014). Several signalling associated proteins were encoded by above mentioned genes such as ras-related protein RABC1 it is a GTP-binding protein that regulates stomatal movements and drought stress responses by mediating the interaction with ABA (Wu et al., 2015), calcium mediated signalling acquisition of stress response (Jiao et al., 2017; Li, 2020), auxin-responsive protein SAUR22 that is known to promote cell expansion (Ren and Gray, 2015), calcium-binding EF hand family protein which actively bind to Ca^2+^ and chelate the cytosolic Ca to regulate Ca homeostasis (Mohanta et al., 2015). Tr-type G domain-containing protein which control a multitude of biological processes, ranging from cell division, cell cycling, and signal transduction, to ribosome assembly and protein synthesis (Jin et al., 2021). The above-mentioned genes exhibited tissue-specific expression, with maximum expression observed during the seed-filling stage, while fewer genes were expressed during the anther development.

During the synteny analysis, for MQTL1.4, the maximum number of orthologs were present on chromosome 9 in rice and on chromosomes 5 and 7 in wheat. These conserved genes encode major transcription factors and enzymes that play important roles in major metabolic pathways. For MQTL2.4 and 2.8, rice orthologs were found on chromosome 4 and on chromosome 2 in wheat, respectively. MQTL6.4 showed conserved sequences on chromosome 6 in rice and chromosome 7 in wheat. In MQTL6.8, orthologs were present on chromosome 5 in rice and on chromosomes 1 and 7 in wheat. In MQTL8.1, orthologs accumulated on chromosomes 3, 4, 5, and 7 in wheat, and on chromosomes 6, 3, and 1 in rice. In MQTL10.8, conserved sequences were reported on chromosome 4 in rice and chromosome 2 in wheat. Overall, chromosomes 1, 2, 3, 4, 5, and 7 exhibited conserved regions in wheat for quality and yield-associated traits, with the maximum number of genes on chromosome 7 (Liu et al., 2020; Soriano et al., 2021; Arriagada et al., 2022; Gudi et al., 2022; Khan et al., 2022). In rice, conserved genes were reported on chromosomes 1, 3, 4, 5, 6, and 9, with maximum synteny on chromosomes 4 and 6 (Kulkarni et al., 2020; Khahani, et al., 2021; Zhang et al., 2021; Aloryi et al., 2022; Kumar R. et al., 2022). Therefore, these validated BF-MQTLs, showing synteny relations between different cereal crops, are believed to be promising for improving yield potential and developing high-quality maize varieties through marker-assisted breeding programs.

## 5 Conclusion

The present study successfully compiled the results of various mapping studies on yield and quality-related traits. A total of 68 MQTLs and 7 singletons were identified in the current study. More than half of the MQTLs were validated through GWAS, and a total of 10 MQTLs were defined as BF-MQTLs based on their CI and PVE values. A comprehensive analysis revealed nearly 60 different genes within the genomic regions of these BF-MQTLs. The putative genes analysed in this study encode essential enzymes involved in multiple metabolic pathways and transcription factors that impact the regulation of key genes. Additionally, some genes were found to be expressed in chloroplasts and peroxisomes, while others function as transporters and signal proteins. Four potential BF-MQTLs were identified to significantly influence both maize yield and grain nutrient quality, making them highly recommended for further utilization in marker-assisted breeding. The overall findings from this study provide valuable insights into robust genomic regions that can be targeted in future investigations to enhance maize yield and nutrient quality.

## Data Availability

The datasets presented in this study can be found in online repositories. The names of the repository/repositories and accession number(s) can be found in the article/Supplementary Material.

## Supplementary material

SUPPLEMENTARY FIGURE S1 An overview of number of GWAS validated MQTLs associated with different traits.
