# Corrigendum: Unravelling the genetic framework associated with grain quality and yield-related traits in maize (Zea mays L.)

**DOI:** 10.3389/fgene.2023.1343846

**Published:** 2023-12-12

**Authors:** Mehak Sethi, Dinesh Kumar Saini, Veena Devi, Charanjeet Kaur, Mohini Prabha Singh, Jasneet Singh, Gomsie Pruthi, Amanpreet Kaur, Alla Singh, Dharam Paul Chaudhary

**Affiliations:** ^1^ Division of Biochemistry, Indian Institute of Maize Research, Ludhiana, Punjab, India; ^2^ Department of Plant Breeding and Genetics, Punjab Agricultural University, Ludhiana, Punjab, India; ^3^ Department of Basic Sciences and Humanities, Punjab Agricultural University, Ludhiana, Punjab, India; ^4^ Department of Floriculture and Landscaping, Punjab Agricultural University, Ludhiana, Punjab, India; ^5^ Agricultural and Environmental Sciences, McGill University, Montreal, QC, Canada; ^6^ Department of Biotechnology, Punjab Agricultural University, Ludhiana, Punjab, India

**Keywords:** maize, yield, quality, meta-QTLs, breeder-friendly, candidate genes

In the published article, a **Funding** statement was erroneously not added. The correct **Funding** statement should read as follows, “We acknowledge the funds received from the DST funded Women Scientist Project (DST/WOS-A/LS-197/2021).”

The authors apologize for this error and state that this does not change the scientific conclusions of the article in any way. The original article has been updated.

