# Supplementary figures and images for: Unravelling the genetic framework associated with grain quality and yield-related traits in maize (Zea mays L.)
Source: Front Genet. 2023 Aug 7;14:1248697. doi: 10.3389/fgene.2023.1248697 (PMC10440565; doi:10.3389/fgene.2023.1248697)

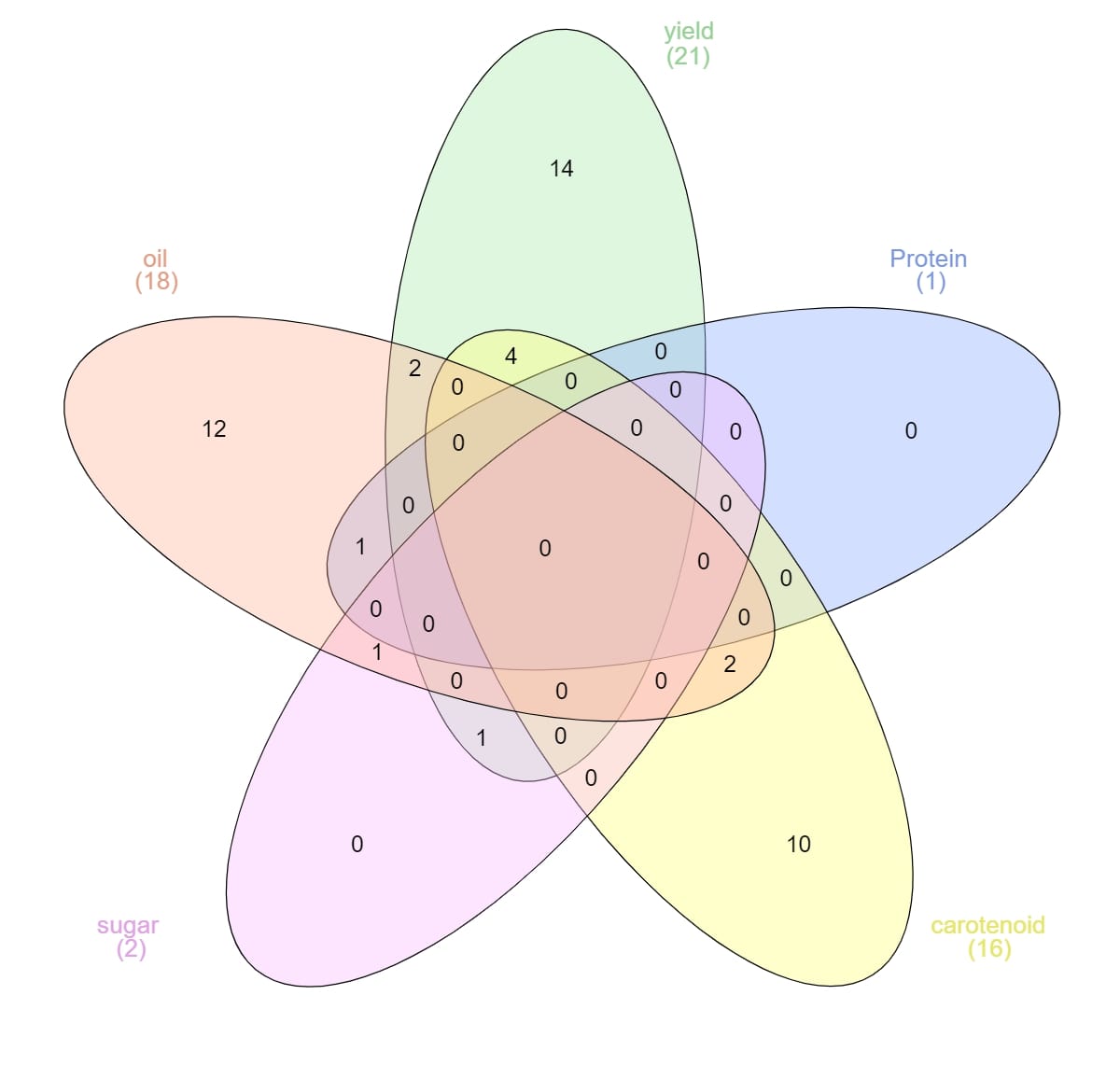

Supplement: Supplementary file 3 [file Image1.jpeg]
